# Peer Review of “COVID-19 and Cybersecurity: Finally, an Opportunity to Disrupt?”

**DOI:** 10.2196/29070

**Published:** 2021-05-06

**Keywords:** COVID-19, cybersecurity, challenges and disruption, data protection, privacy, health data

## Round 1 Review:

This paper [1] presents a viewpoint on the cyber threat issues in the context of COVID-19. It is well written and comprehensive. The scope matches the journal and is of interest to the challenging topic of privacy in contact tracing. However, there is a lack of new evidence or new conclusions. The reported alerts and the discussions are normative and minimal; most of them have already been widely discussed in newspapers and mainstream media.

However, the topic of cybersecurity and privacy will never be outdated since the evolution of COVID-19. The authors must work a lot more to identify the knowledge gap in the existing literature first and then propose novel viewpoints to bridge the gaps. Before drawing a conclusion, the authors must add a discussion on how their findings compare to other studies (references) that were published very recently.

Appropriate references to related work are not covered sufficiently in the list, especially for the contact tracing section. A systematic review of the global deployment status of contact tracing apps is recommended, as in [2].

In addition to the majority of qualitative descriptions, quantitative statistics are suggested to complement and support the statements in the manuscript.

## Round 2 Review:

After the first round of review, the reviewer appreciates the revision effort the authors have made in improving the manuscript.

However, the authors may need to redefine the contribution and the title in a more specific and unique manner.

The current title looks quite broad and not new, as a number of similar articles have been disseminated on the web (eg, [3,4]).

The authors might consider reducing the overlap with published work as illustrated above.
